# 3‐D substructure search by transitive closure in AlphaFold database

**DOI:** 10.1002/pro.70169

**Published:** 2025-05-22

**Authors:** Hao Liu, Aleksi Laiho, Petri Törönen, Liisa Holm

**Affiliations:** ^1^ Organismal and Evolutionary Biology Research Program, Faculty of Biological and Environmental Sciences University of Helsinki Helsinki Finland; ^2^ Institute of Biotechnology HiLIFE, University of Helsinki Helsinki Finland

**Keywords:** Dali, Foldseek, Pfam, protein space, superfamily

## Abstract

Identifying structural relationships between proteins is crucial for understanding their functions and evolutionary histories. We present ISS_ProtSci, a Python package designed for structural similarity searches within the AlphaFold Database v2 (AFDB2). ISS_ProtSci incorporates DaliLite to identify geometrically similar structures and uses a transitive closure algorithm to iteratively explore neighboring shells of proteins. The precomputed all‐against‐all comparisons generated by Foldseek, chosen for its speed, are validated by DaliLite for precision. Search results are annotated with metadata from UniProtKB and Pfam protein family classifications, using hmmsearch to identify protein domains. Outputs, including Dali pairwise alignment data, are provided in TSV format for easy filtering and analysis. Our method offers a significant improvement in recall over existing tools like Foldseek, especially in detecting more distantly related proteins. This is particularly valuable in structurally diverse protein families where traditional sequence‐based or fast structural methods struggle. ISS_ProtSci delivers practical runtimes and flexibility, allowing users to input a PDB file, define the minimum size of the common core, and evaluate results using Pfam clans. In evaluating our method across 12 test cases based on Pfam clans, we achieved over 99% recall of relevant proteins, even in challenging cases where Foldseek's recall dropped below 50%. ISS_ProtSci not only identifies closely related proteins but also uncovers previously unrecognized structural relationships, contributing to more accurate protein family classifications. The software can be downloaded from http://ekhidna2.biocenter.helsinki.fi/ISS_ProtSci/.

## 1 INTRODUCTION

The rapid expansion of protein structure databases, notably the AlphaFold Database (AFDB2), offers unparalleled opportunities to enhance our knowledge of protein function, evolution, and classification (Jumper et al., 2021; Varadi et al., 2021). However, efficiently searching these vast datasets for structurally similar proteins remains a challenge. While current methods (Edgar, 2024; Liu et al., 2024; van Kempen et al., 2024) enable fast structural searches, they often sacrifice recall, limiting their ability to detect more distantly related structures.

Hierarchical classification schemes, such as SCOP (Chandonia et al., 2022), categorize protein structures based on structural and evolutionary relationships. A fold defines the spatial arrangement of secondary structure elements within a domain, forming a characteristic pattern. Superfamilies group domains with structural or sequence features suggesting common ancestry, even without significant sequence similarity, while families represent more closely related proteins within a superfamily. The common core refers to structural elements consistently present across all instances of a given fold class.

In this study, we introduce a transitive closure approach to structural similarity search, which works similarly to web crawling by connecting related structures through a chain of intermediate links. This approach identifies indirect relationships by linking structures not just directly but also through shared connections, broadening the scope of similarity detection. In web crawling, search engines begin with a set of known web pages (starting points) and follow links to discover additional content. The crawler iteratively explores deeper layers of links, gradually expanding its search space and mapping large portions of the web. Similarly, in our transitive closure search for 3‐D structural similarity, the algorithm starts with a query protein structure and identifies “neighboring” structures that share some degree of similarity. Rather than stopping at directly related structures, the search iteratively expands to explore neighbors of neighbors, progressively broadening the search space.

**FIGURE 1:**
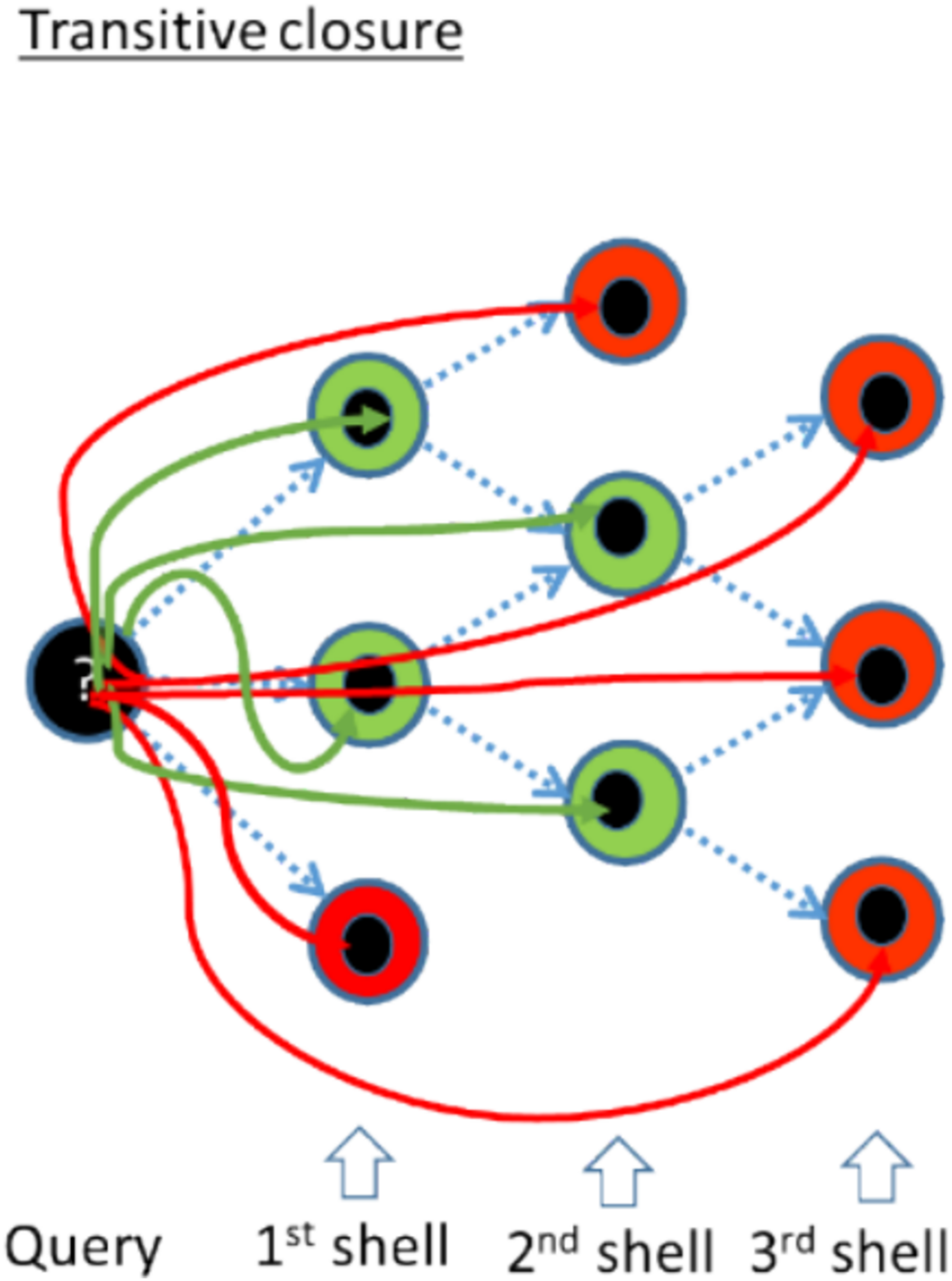
Schematic representation of the transitive closure search method. The goal is to efficiently identify all protein structures with significant geometrical similarity to the Query structure, as validated by DaliLite. Our method iteratively explores neighboring shells in protein space, capturing distant structural relationships that might be missed by single‐step searches. Dashed blue arrows represent neighbor relationships predicted by Foldseek. Solid arrows are color‐coded based on the validation results: green indicates pairs that pass the filtering criteria (Dali Z‐score and alignment length), while red indicates pairs that fail. The search terminates when no new candidates in a shell meet the validation criteria.

Given the immense scale of both the web and protein networks, a precise and exhaustive traversal search is impractical. A key element of our method is the two‐tiered evaluation of structural similarities: the initial neighbor graph is generated using a fast but imprecise method (Foldseek), which is well‐suited for large‐scale comparisons (van Kempen et al., 2024). As the search progresses, new candidates in each neighbor shell are validated using DaliLite, a slower but more accurate tool for structural alignment (Holm, 2019). This validation step ensures that reported results include only proteins that share geometrical similarity with the query structure and that non‐productive search paths are pruned. This approach enables the discovery of distantly related protein structures that may not be apparent in the initial search. Just as web crawlers uncover deeper, non‐obvious connections within the web, transitive closure reveals the full spectrum of structural relationships between proteins, including more distant relatives (Figure 1).

We validate the effectiveness of our approach by using Pfam (Mistry et al., 2021) clans or SCOPe (Chandonia et al., 2022) folds as ground truth references and benchmarking against Foldseek, one of the fastest available structural search tools. Our results clearly demonstrate that the transitive closure method offers a significant improvement in recall, particularly for diverse protein families where existing methods often fall short. Beyond its superior recall, our approach also delivers practical runtimes with carefully selected input parameters, making it highly applicable for targeted, in‐depth studies. These findings underscore the potential of transitive closure to provide a more sensitive and robust tool for structural similarity searches, offering deeper insights into protein function and evolution within the rapidly expanding realm of protein databases.

## 2 ILLUSTRATIVE EXAMPLES

**FIGURE 2:**
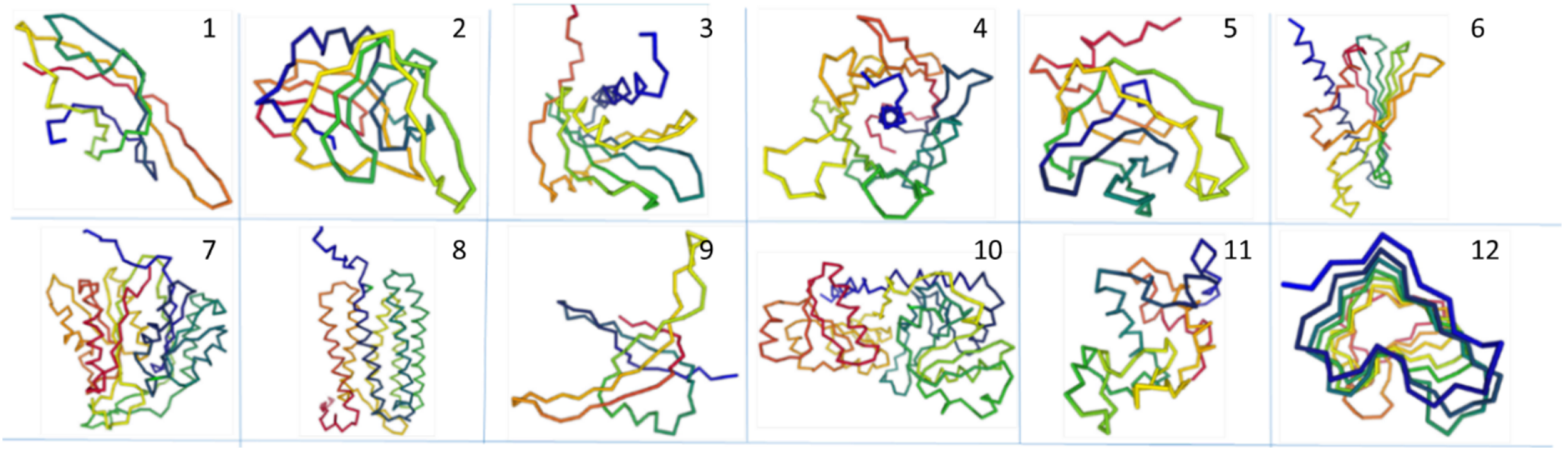
Cartoon representations of the query structures for the 12 test cases of Table 1.

To test the hypothesis that transitive closure in a fold‐space graph can effectively recall all instances of a fold type in the database, we applied ISS_ProtSci to 12 test cases (Figure 2), using Pfam clans as positive controls (Table 1). Examples 1–7, representing Pfam clans (superfamilies) of varying sizes, were relatively straightforward. In contrast, Examples 8–12, drawn from well‐established literature cases (Holm & Sander, 1994a; Jenkins et al., 1998; Liu & Mushegian, 2003; Murzin, 1993; Yang et al., 2021), presented greater challenges, providing a more rigorous test of transitive closure and Foldseek while highlighting their performance differences.

**TABLE 1:** Test set with user inputs and runtime for search.

Case #	Query	Core size	*Q*_cov_ (%)	Pfam clan	*Z*_median_	Clan description	Search time (s)
1	3sb3A	85	84	CL0524	5.8	MPT63‐MPB63	64
2	3zg4A	100	78	CL0508	11.4	l,d‐transpeptidase catalytic domain	19
3	2mj7A	85	60	CL0545	11.0	Clathrin (AP) and COPI appendage platform subdomain	996
4	amseA	120	63	CL0395	13.4	Tubby C‐terminal domain‐like	52
5	ezjrA	40	33	CL0080	5.8	Beta‐tent fold	152
6	fbuzA	100	58	CL0209	9.8	Bet v 1 like	964
7	jw11A	100	34	CL0046	21.7	Thiolase‐like Superfamily	676
8	mr59A	180	75	CL0192	8.7	Seven transmembrane receptors (Yang et al., 2021)	2271
9	k0wxA	50	52	CL0021	3.9	OB‐fold (Murzin, 1993)	2430
10	rfhrA	200	63	CL0113	12.7	GT1 (Liu et al., 2024)	619
11	lx55A	60	47	CL0037	7.4	Lysozyme (Holm & Sander, 1994a)	230
12	1ee6A	160	81	CL0268	12.9	Right‐handed beta helix similar to that first found in pectate lyase (Jenkins et al., 1998)	625

*Note*: Query identifiers are internal to DaliLite. *Q*_cov_ is the core size divided by the length of the query. *Z*_median_ is the median Z‐score of the Dali alignment of the query structure to the TRUE_1 set.

In designing these experiments, we carefully selected both the query structure and common core size. A “bare‐bones” query structure—containing only essential elements—was chosen to avoid extraneous features that could mislead the search. The common core size parameter also played a crucial role: if set too small, the transitive search could continue indefinitely without producing relevant matches; if too large, the search might terminate prematurely, missing biologically meaningful results. Transitive closure searches averaged 13 min of wall‐clock time, with a median of 10 min (Table 1), making them practical for routine use. However, since runtime is highly sensitive to input parameters, ISS_ProtSci is provided as a standalone tool rather than a web server.

### 2.1 Completeness of search results

In principle, our transitive closure algorithm will semi‐deterministically return all database proteins that match the query structure with the required common core size, provided these proteins are connected in the underlying neighbor graph. The semi‐determinism arises from the selective sampling of representatives among closely related database proteins. However, from a user's perspective, it is important to have some assurance of the result set's completeness. To address this, we used Pfam clans as positive controls. The Pfam classification (Mistry et al., 2021), which covers approximately 70% of all proteins in the UniProt database (from which AFDB2 is derived), is expert‐curated, ensuring the biological relevance of the clans.

We report three series of evaluation results, corresponding to clan member proteins detected by hmmsearch using the trusted cutoff (suffix _0), additionally requiring geometrical similarity resulting in Dali Z‐scores above 2 (suffix _1), and further requiring that the Dali alignment length exceeds the minimum size of the common core (suffix _2). The relatively small differences between the protein counts in columns TRUE_0, TRUE_1, and TRUE_2 in Table 2 indicate that biologically relevant matches are generally also geometrically similar and that the chosen core size (an input parameter, Table 1) was appropriate. Using the most stringent reference set, TRUE_2—filtered by both geometrical similarity and alignment length—we are pleased to observe that the transitive closure algorithm retrieved over 99% of relevant matches in ten out of twelve cases and over 85% in the remaining two (Supplementary Table 3). Except for cases 1, 3, 6, and 7, the filtered output set (P_2) is also less than double the size of TP_2, providing a valuable starting point for more in‐depth study and potentially expanding the Pfam clan (Table 2).

**TABLE 2:** Impact of result filtering on evaluation counts for test cases.

#	Reference			Closure					Foldseek (e‐value <1)					
	TRUE_0	TRUE_1	TRUE_2	P_0	P_1	P_2	TP_1	TP_2	P_0	P_1	P_2	TP_0	TP_1	TP_2
1	15	14	13	840	766	269	14	13	571	444	28	14	14	13
2	74	73	64	184	79	65	72	64	1497	78	65	72	72	64
3	186	184	181	80,357	11,752	5171	183	181	54,559	3752	2086	179	179	178
4	359	342	300	1354	494	445	315	299	1826	682	399	319	317	285
5	740	719	719	8645	794	794	**613**	**613**	459	395	395	325	320	320
6	1308	1289	1213	40,253	13,391	2618	1274	1211	4449	3514	1448	1172	1166	1144
7	2058	1978	1942	36,030	9308	5783	1965	1939	1975	1920	1895	1892	1868	1866
8	10,300	9387	5568	33,701	13,274	6012	**9209**	**5564**	3946	3637	2226	3597	3352	2093
9	18,280	10,603	10,299	78,555	12,302	11,982	**10,538**	**10,236**	1511	1404	1404	1363	1269	1269
10	4589	4489	3656	8390	5386	3763	**4079**	**3652**	8728	8632	2330	2411	2411	2282
11	1100	720	718	2329	664	661	**625**	**624**	228	228	227	222	222	221
12	1753	1732	1210	17,735	3162	1356	**1417**	**1204**	638	638	544	571	571	517

*Note*: Transitive closure recall at least 60% higher than Foldseek recall is shown in bold type. Suffixes (_0, _1, _2) represent different filtering levels (see Section 5.5). Precision‐recall analysis is shown in Figure 4.

Abbreviations: TRUE, reference; P: Positives; TP, true positives.

Due to the unequal size distribution of Pfam families, counting proteins as done in Table 2 could potentially introduce bias. However, this does not appear to be the case, as the transitive closure algorithm successfully covered the diverse member families (Supplementary Table 2).

Pfam clans vary in their characteristics: some consist of closely related families, while others are highly diverse. In cases where Foldseek performs well, transitive closure can offer little improvement—Foldseek recall (TP_2) is within 6% of that of transitive closure in cases 1–4 and 6–7. However, in the more challenging cases 5, 8, 9, 11, and 12, Foldseek recall drops below 50%. In contrast, transitive closure with alignment length filtering achieves over 99% recall of the reference set in 9 out of 12 cases, compared to just two for Foldseek. These results demonstrate that transitive closure is a more sensitive search tool than Foldseek, particularly for challenging cases.

### 2.2 Quality of matches

**FIGURE 3:**
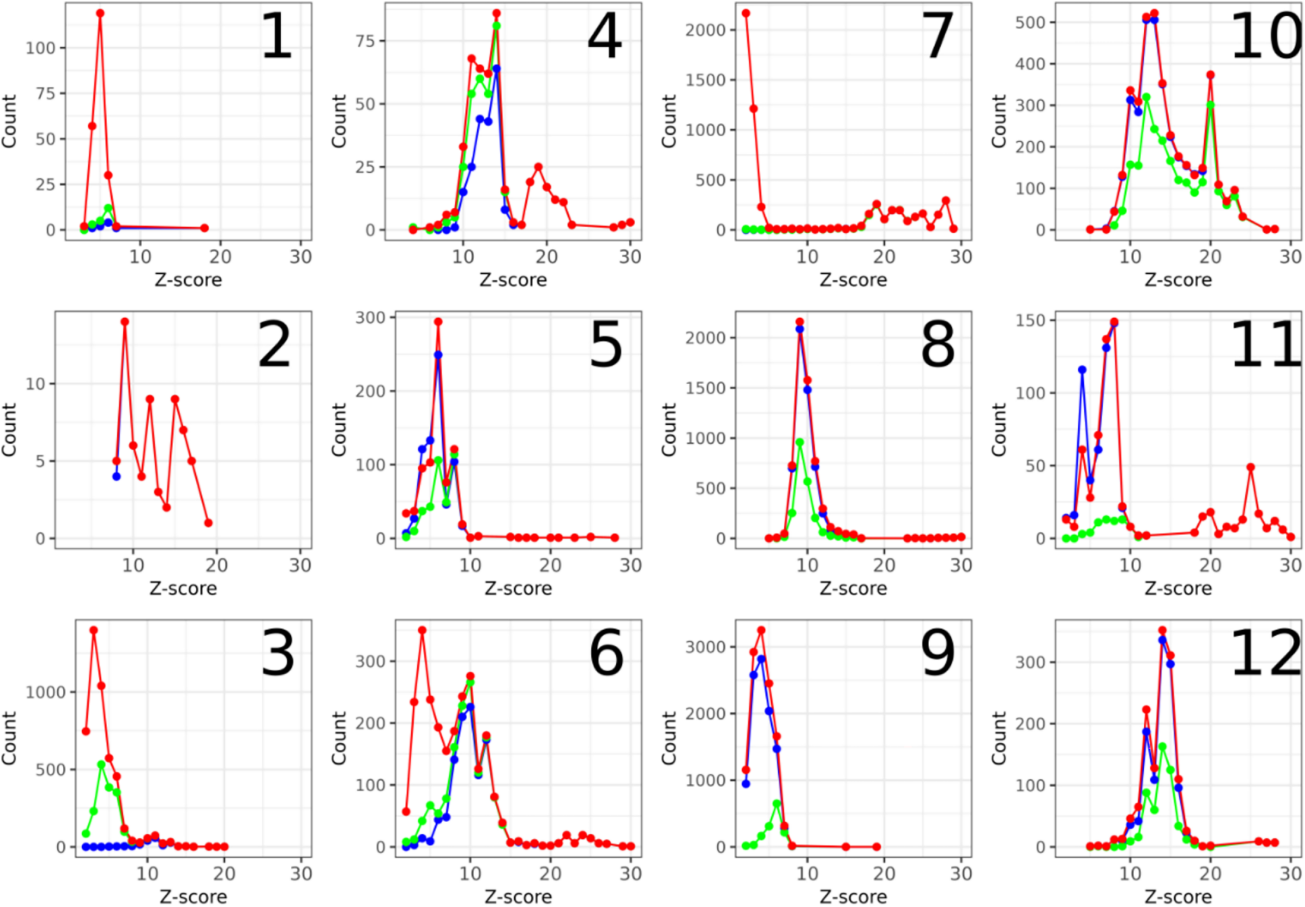
*Z*‐score distributions for the P_2 set of cases 1–12 for transitive closure (red), Foldseek first shell (green), and Pfam clan (blue).

The quality of matches is validated using Dali. A comparison of Dali *Z*‐score distributions from transitive closure and Foldseek against a baseline of annotated Pfam clan members shows that both methods capture strong structural matches with high *Z*‐scores (Figure 3). However, in cases 5 and 8–12, transitive closure outperforms Foldseek in detecting Pfam clan members. Additionally, in cases 4–6 and 8–12, transitive closure identifies a number of extra matches with *Z*‐scores typical of Pfam clan members, suggesting potentially relevant but unannotated clan relationships.

While transitive closure's *Z*‐score distribution closely mirrors that of the reference classification in most cases, Foldseek's performance declines noticeably in cases 5, 9, and 11. Table 1 highlights a shared characteristic among these cases: they contain particularly small structural cores—both in terms of query coverage (<60%) and absolute size—along with lower median *Z*‐scores (<8) among clan members in the reference classification. This pattern suggests a higher degree of structural divergence within these clans. When clan members exhibit lower structural similarity, Foldseek struggles to distinguish them from unrelated proteins.

ISS_ProtSci includes a configurable *Z*‐score threshold, but since optimal cutoffs vary across protein families, we opted to accept all Dali‐reported hits (*Z* > 2). Notably, cases 1, 3, 6, and 7 exhibit a peak of apparent false positives with low *Z*‐scores, indicating weak structural matches. Dali performs local structural alignments across entire proteins, normalizing scores based on domain size. For *Z*‐score calculations, it applies an internal domain decomposition algorithm (Holm & Sander, 1994b), assigning the match to the most representative candidate domain pair. Lower *Z*‐scores typically arise from partial domain matches, which occur in three scenarios: (1) the subject domain is smaller and contained within the query domain, (2) the query domain is smaller and contained within the subject domain, or (3) only portions of either domain align structurally. Since we filtered for query coverage, these apparent false positives likely represent partially aligned larger subject domains (Supplementary Figure 1).

### 2.3 Precision‐recall evaluation

It is natural that the broader search enabled by transitive closure improves results by identifying more relevant matches. The key question is at what cost this improvement is achieved. In bioinformatics, performance criteria used to rank binary classifiers (positive vs. negative) include precision‐recall and *F*_max_ analysis. Here, we use Pfam clans (superfamilies) as the ground truth, as this reference classification is available for proteins in AFDB2. It is important to note that the precision values should be considered provisional, since Pfam assigns only positive cases, and ~30% of proteins in the database remain unlabeled.

**FIGURE 4:**
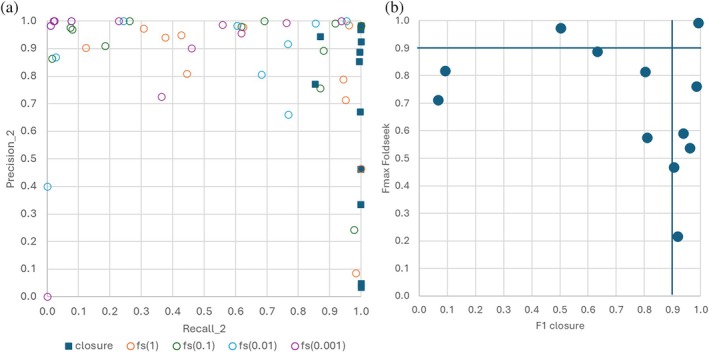
(a) Precision and recall values of Supplementary Table 3 for the end point of transitive closure search (blue squares), and for several e‐value cutoff points for direct Foldseek (fs) search (open circles). (b) F1‐scores of the P_2 sets by transitive closure and the maximum F1‐score of Foldseek at any e‐value cutoff.

F1 scores were compared for both the transitive closure and Foldseek methods across twelve test cases. We evaluated precision and recall at the end point of transitive closure (with filtering). Foldseek produces a ranked list, which can be cut at various points (Supplementary Table 3). *F*_max_ is the best F1 score over all cut points. Where transitive closure excels in recall, Foldseek delivers high precision (Figure 4a). In five cases, Foldseek achieves better F1 scores, especially where its strict e‐value cutoffs capture nearly the entire clan. In these cases, expanding the search with transitive closure lowers precision without significantly improving recall. In six cases, transitive closure outperforms Foldseek, especially in the more diverse clans. Transitive closure achieves an F1 score (*F*_max_) > 0.9 in six cases, compared to only two such cases for Foldseek (Figure 4b).

Recall is our primary focus, as retrieving all relevant matches is crucial for comprehensive analysis. Transitive closure excels in this regard, allowing us to capture distant relationships. While precision can be improved through post‐processing filters to eliminate false positives, false negatives pose a greater challenge since they are irretrievably lost from further analysis. The Dali alignment length filter effectively balances this, ensuring an acceptable level of precision without sacrificing recall. Further, if positive controls are available, they can be used to adjust a *Z*‐score cutoff for classification.

## 3 BENCHMARKING ON A LARGE‐SCALE SCOPe DATASET

To further assess search performance, we conducted a large‐scale benchmark using SCOPe as the reference classification. Applying the default core size, we tested a query set of 93 SCOPe domains. The results reinforce the conclusions drawn from our initial set of 12 test cases.

The SCOPe classification allows for a detailed evaluation of structural divergence effects on search performance. Transitive closure achieves higher recall than Foldseek at all classification levels (Table 3). The difference is relatively small at the family level, with transitive closure outperforming Foldseek by 7 percentage points. However, the gap widens to 15 percentage points—at the family‐to‐superfamily transition, where sequence similarity weakens. Foldseek's performance declines even more sharply at the superfamily‐to‐fold transition, where sequence similarity is largely absent and structural divergence becomes more pronounced.

**TABLE 3:** Comparison of recall_1 (TP_1/TRUE_1) by transitive closure and Foldseek in SCOPe benchmark at different levels of the reference classification.

Recall_1 statistic	Level	Closure	Foldseek(e < 1)
Pooled	All pairs	0.74	0.39
	Fold pairs	0.76	0.28
	Superfamily pairs	0.59	0.40
	Family pairs	0.92	0.77
Query‐wise average	All pairs	0.80	0.59
	Fold pairs	0.56	0.23
	Superfamily pairs	0.76	0.51
	Family pairs	0.96	0.89
Query‐wise median	All pairs	1.00	0.63
	Fold pairs	0.74	0.13
	Superfamily pairs	1.00	0.55
	Family pairs	1.00	1.00

*Note*: Fold pairs (*n* = 80,116) are in the same SCOPe fold but different superfamilies, superfamily pairs (*n* = 41,419) are in the same SCOPe superfamily but different families, and family pairs (*n* = 23,460) are in the same SCOPe family.

**FIGURE 5:**
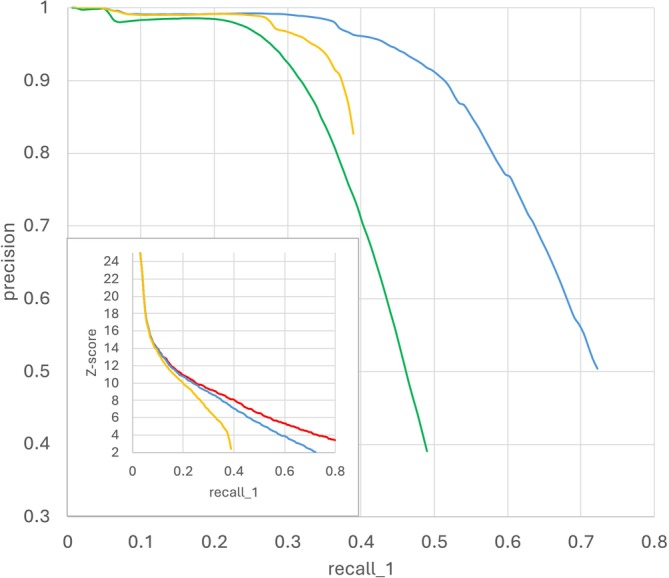
Precision‐recall curves for transitive closure results ranked by *Z*‐score (blue curve), Foldseek direct hits up to e‐value 1 ranked by *Z*‐score (orange curve), and Foldseek direct hits up to e‐value 10 ranked by e‐value (green curve). Pairs assigned to the same SCOPe fold were considered correct. The inset shows the *Z*‐score cutoff as a function of recall for transitive closure (blue curve), Foldseek ranked by *Z*‐score (orange curve), and the SCOPe reference (red curve).

A precision‐recall analysis further underscores the advantage of transitive closure (Figure 5). The area under the precision‐recall curve (AUPRC) is consistently higher for transitive closure than for Foldseek, indicating superior ranking of relevant results. We also find that Foldseek hits with e‐values between 1 and 10 contribute little to overall search performance. Performance trends also vary by fold class (Supplementary Table 6). Both methods struggle with all‐alpha folds, while the best performance is seen for alpha/beta folds. Transitive closure retains high performance across all‐beta and alpha+beta folds, whereas Foldseek's performance declines for alpha+beta folds. Finally, we note that pooling all results across queries for AUPRC analysis is unfavorable, with notable differences to query‐wise AUPRC values (Supplementary Table 6). On average, query‐wise AUPRC for Transitive Closure is 7 percentage points higher than the corresponding pooled AUPRC, while Foldseek's query‐wise AUPRC exceeds the pooled value by 16–19 percentage points. This suggests that ranking search results by Dali *Z*‐score (or Foldseek e‐value) is effective within individual query sets, but scoring scales differ across queries (Holm, 2019).

Search runtimes correlate linearly with the number of validation tests (Supplementary Figure 2). The distribution of runtimes is relatively uniform on a log scale, suggesting that excessively long searches are exponentially rare. In the SCOPe benchmark, the maximum search time for a query was 7203 s, while the average was 784 s, and the median was 188 s. Transitive closure performed 3.5 million Dali validation tests, yielding 234,427 hits in the P_1 set, while the direct Foldseek search resulted in 266,000 Dali validation tests, yielding 76,615 hits in the P_1 set.

## 4 DISCUSSION

Overall, transitive closure enables a more comprehensive structural search, achieving high recall while maintaining practical runtimes. Its flexibility and sensitivity make it a powerful tool for studying protein fold families, refining existing fold classifications, exploring evolutionary relationships, and generating multiple structural alignments for deeper analysis.

However, the ability to perform comprehensive searches introduces a new challenge: managing large result datasets. Pfam labels offer a practical solution for reducing redundancy in these outputs. While structural comparisons are not required to retrieve known Pfam members—since they were originally grouped using sequence profile models—they provide more precise structural alignments, which can serve as seeds for refined profile models. Furthermore, search results that appear as “false positives” may instead highlight novel relationships, particularly when unclassified queries probe new regions of protein space.

In the following, we demonstrate the analysis of novel findings using test set case #4 as an example.

### 4.1 Re‐evaluation of protein family classification

**FIGURE 6:**
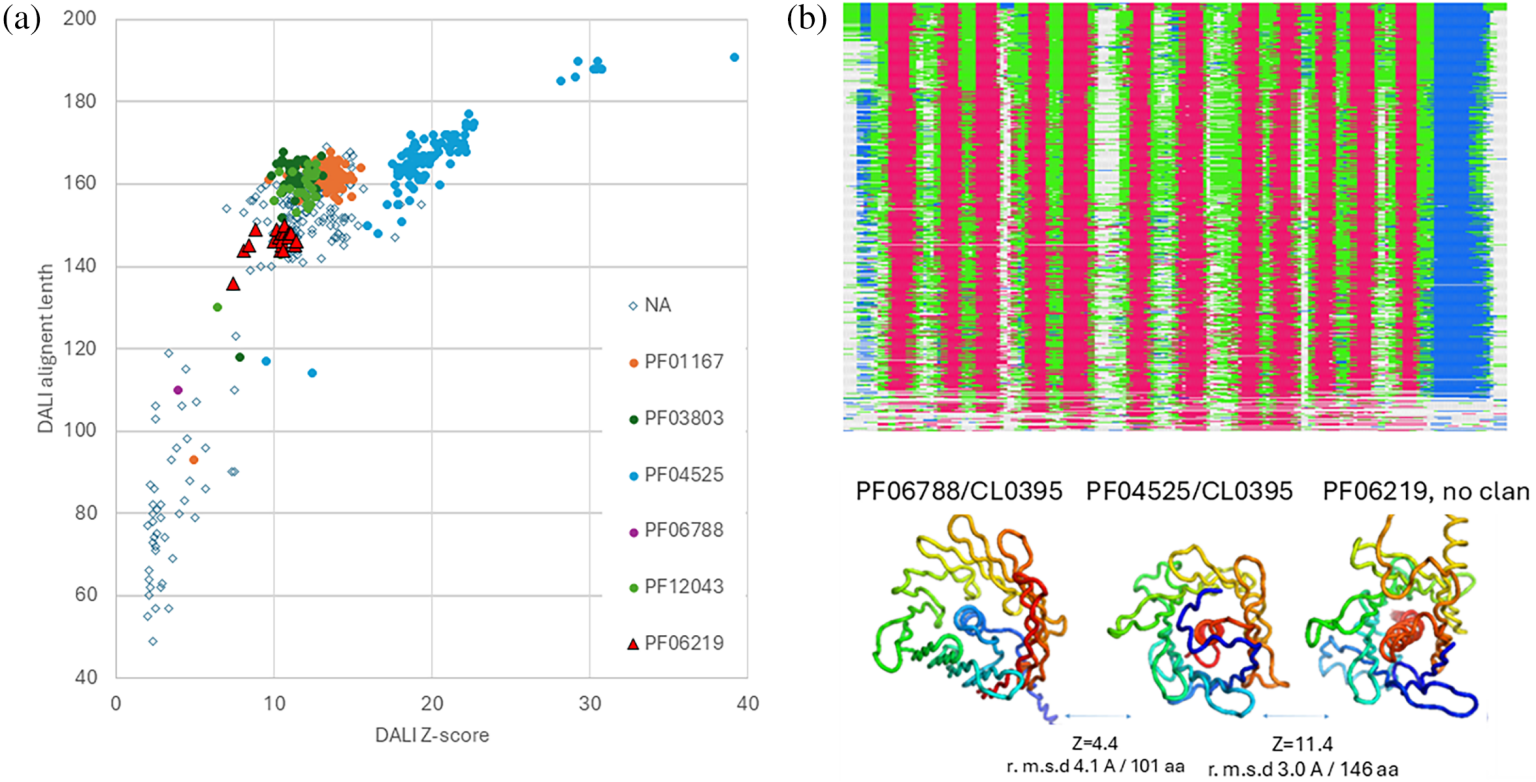
Visualization of large result sets. (a) The search results for benchmark case #4 revealed an outlier family (PF06788, magenta) and a newly identified addition (PF06219, red triangles) to Pfam clan CL0395 (filled circles). (b) Stacked alignment for test set case #4, colored by secondary structure (red: beta strands; blue: alpha helix; green: loop; gray: unaligned). The data is seriated by maximizing the similarity of secondary structure assignments between adjacent rows. Rendered with msaviewer (Yachdav et al., 2016). (c) Pfam family representatives u383A (apparent false negative), amseA (query structure), and g8jA (apparent false positive) were visualized using PyMOL (Schrödinger & DeLano, 2020). Structures are colored from blue at the N‐terminus to red at the C‐terminus in a rainbow gradient.

Visualizing the Dali validated matches from the P_1 set in a *Z*‐score versus alignment length plot, annotated with known Pfam families, offers a comprehensive overview of the results (Figure 6a). Stacked alignment strings can be extracted from the output file, converted to FASTA format, and viewed in a standard multiple sequence alignment viewer. For example, the chosen query structure for test set case #4 effectively represents the common core (Figure 6b). This alignment demonstrates that hundreds of database proteins can be accurately aligned in three dimensions, preserving all components of the 12‐stranded beta barrel, which features a helix at its center (Figure 6c).

Test set case #4 belongs to the Tubby C‐terminal domain‐like clan. We identified a previously unrecognized family, PF06219, which displayed strong *Z*‐scores and sufficient alignment length to meet our common core requirement (Figure 6a). This Pfam family actually represents a plant‐specific fusion of a Tubby‐like domain (Bateman et al., 2009) and a C2 domain (Zhang & Aravind, 2010), whose functions—acting as a transcription factor and membrane tether—are well aligned with its role in regulating root elongation (Tian et al., 2022). In contrast, family PF06788 has been assigned to the clan, but it exhibited poor *Z*‐scores and alignment lengths, suggesting structural divergence from other clan members. Notably, the representative AlphaFold model for PF06788 features a 16‐stranded beta barrel instead of the expected 12‐stranded barrel, along with a topological permutation of the helix from the C‐terminus to the N‐terminus (Figure 5c). PF06788 is an uncharacterized protein family (UPF0257) found in *Enterobacteria*. The closest structural neighbors of PF06788 in the AFDB2 database belong to another clan entirely, namely, clan CL0193 of beta barrel membrane proteins, and particularly family PF00267 of Gram‐negative bacterial porins (Supplementary Figure 3). This example illustrates how our approach effectively highlights both novel discoveries and instances of structural variation within Pfam clans.

### 4.2 Scope of application

Our approach is best suited for single‐domain queries, particularly those that are small, compact, and central to their protein family. When the query represents a single domain, all database matches correspond to that same domain, simplifying analysis and classification. Users can identify domains within a large query structure through various methods, such as visual inspection with molecular graphics software like PyMOL or automated domain detection tools (Lau et al., 2024; Zhang et al., 2023). Alternatively, the Dali web server (http://ekhidna2.biocenter.helsinki.fi/ISS/) offers a time‐limited database search, generating a seriated graphical overview (similar to Figure 5 but for a multidomain query) that highlights recurrent domains as rectangular blocks. It is worth noting that while the query structure should preferably be a single domain, the AFDB2 target database consists of complete proteins, and multi‐domain targets are handled transparently since Dali performs local alignments.

By leveraging transitive closure, our method goes beyond direct query‐based searches, improving the detection of remote homologs and structurally similar folds. Benchmarking against Pfam and SCOPe confirmed that transitive closure captures more distant relationships than Foldseek alone. To further refine fold‐space mapping in the future, we plan to explore alternative embeddings based on protein language models or sequence‐independent structural features, assessing their potential to bridge connectivity gaps and enhance structural classification.

## 5 MATERIALS AND METHODS

### 5.1 Implementation

ISS_ProtSci is a Python package developed for performing structural similarity searches within the AlphaFold Database v2. It uses Dali (Holm, 2019) for structural alignment to quantify the geometric similarity to a given Query structure. The package integrates Python scripts with external programs and publicly available databases (see Supplementary Table 5). Linux shell scripts are included for a streamlined workflow, which includes (1) performing database searches, (2) annotating results with metadata, and (3) analyzing the completeness of results using a Pfam clan or family as positive controls. The package also includes results from the Pfam and SCOPe evaluation test sets, generated using these scripts.

The only mandatory inputs are a PDB‐formatted query structure and a unique run identifier, which serves as a prefix for the result files. In the transitive closure algorithm, users can optionally specify the minimum core size (minlali) and Dali *Z*‐score cutoff (zcut) to control the search depth. Additionally, a configurable timeout parameter allows automatic termination of searches before completion. Experiments were executed using DaliLite with OpenMPI on 40 CPUs.

The output from ISS_ProtSci includes Dali alignment scores, rotation and translation matrices for 3D superimpositions, UniProt accession numbers, functional descriptions, taxonomic information, Pfam and secondary structure annotations, and stacked structural alignments (Supplementary Table 1). These data can be used to filter results, such as excluding entries lacking a required element of the common core or selecting those with a specific amino acid type at an active site position. Beyond command‐line analysis, Dali identifiers of hits can be input into the Dali server for interactive visualization using the pairwise comparison option (Holm et al., 2023).

### 5.2 Data preprocessing

#### 5.2.1 Protein structure data

Query structures were obtained from the AlphaFold Database v2 (AFDB2) (https://ftp.ebi.ac.uk/pub/databases/alphafold/v2/) or the Protein Data Bank (https://rcsb.org). SCOPe‐40 domain coordinates were downloaded from https://scop.berkeley.edu/downloads/pdbstyle/pdbstyle‐sel‐gs‐bib‐40‐2.07.tgz. Preprocessed AlphaFold structural models for local DaliLite analysis were retrieved from http://ekhidna2.biocenter.helsinki.fi/dali/digest.html.

#### 5.2.2 Foldseek processing

Foldseek was used (i) to identify entry points into the fold‐space graph and (ii) to generate an all‐against‐all structural comparison. The latter was performed using Foldseek's *easy‐search* with parameters ‐e1 and ‐max‐seqs 50,000, completing in 5 days on 80 CPUs. The resulting data is stored in a remote SQLite3 database and accessed by ISS_ProtSci. For entry‐point identification, a preprocessed, indexed database enables Foldseek searches against AFDB2.

#### 5.2.3 AFDB2 metadata

UniProt sequences in FASTA format were downloaded from Uniprot, and metadata (accession, description, taxonomy, and gene symbol) were extracted from the sequence headers of the Fasta files. ISS_ProtSci retrieves this metadata from the remote data server in Helsinki.

#### 5.2.4 Pfam ground truth

The Pfam‐A.hmm profile library and Pfam‐C family‐clan associations were downloaded in September 2024 from https://www.ebi.ac.uk/interpro/download/pfam/. Pfam domains in AFDB2 were identified using *hmmsearch* with the trusted cutoff, yielding 1,729,869 Pfam family assignments across 838,125 AFDB2 proteins, spanning 689 clans and 7,926 families not in clans. Processed Pfam data tables are included in ISS_ProtSci for evaluation purposes.

#### 5.2.5 SCOPe ground truth

Since ISS_ProtSci reports hits to AFDB2 proteins, SCOPe classification labels were assigned via sequence‐based transfer from SCOPe‐2.07's 40% identity‐filtered subset. The domain composition of PDB proteins classified in SCOPe was transferred to AFDB2 proteins if the BLAST (Altschul et al., 1990) alignment covered at least 80% of both the query and target protein, sequence identity exceeded 40% and the E‐value was below 1e‐5. Overrepresentation of closely related proteins was reduced using CD‐HIT (Li & Godzik, 2006) filtering to 70% sequence identity. This resulted in 92,980 AFDB2 proteins being assigned SCOPe labels.

#### 5.2.6 SCOPe benchmark query set

The SCOPe benchmark is based on the 40% identity‐filtered subset of SCOPe‐2.07. A 1% random subsample was taken, excluding artificial classes (e‐l), duplicate family entries, and unclassified families. The final test set comprised 93 queries for AFDB2 searches with ISS_ProtSci (Supplementary Table 4).

### 5.3 Database search algorithm

**FIGURE 7:**
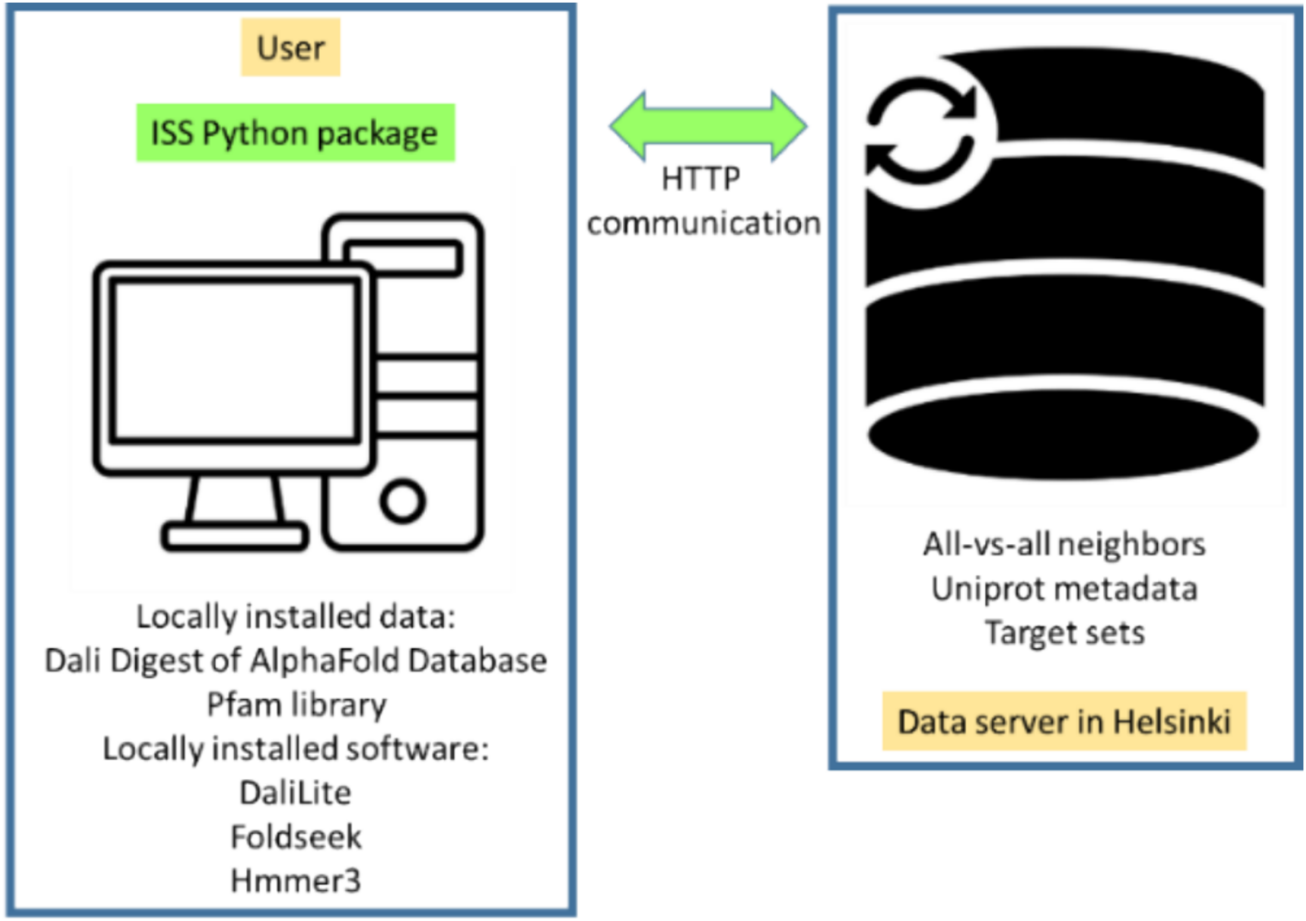
System overview: most computations are done locally on the user's computer, while a huge knowledge base is stored on a remote data server.

The search process follows an iterative transitive closure algorithm (Figure 1) to explore neighboring shells of protein structures, leveraging a precomputed all‐against‐all comparison generated with Foldseek. Foldseek was selected for its speed, despite some imprecision, and its max‐seqs parameter was set to 50,000 to enhance sensitivity. To keep the all‐against‐all database manageable, only protein pairs with an e‐value below 1 were included, as higher e‐values produce an overwhelming number of mostly irrelevant matches. Due to the database's size, ISS_ProtSci accesses it remotely (Figure 7). While Foldseek was used in this implementation, other rapid comparison methods could replace it in future versions.

The all‐against‐all database consists of proteins from AlphaFold Database v2 (AFDB2), but the Query structure itself is not included. The first neighbor shell is identified using Foldseek with a relaxed e‐value cutoff of 1.0, prioritizing sensitivity. In subsequent neighbor shells, a stricter e‐value cutoff of 0.01 is applied to improve precision as the search expands. This strategy ensures high initial sensitivity while maintaining specificity in later stages for efficient identification of related structures.

Each iteration of the search involves a batch of DaliLite comparisons to identify new geometrically similar proteins. The search depth is controlled by two parameters: (1) zcut, the minimum Dali Z‐score required for a match (fixed at 2.0 in this study) and (2) minlali, the minimum Dali alignment length, set manually for the Pfam benchmark and left at its default for the SCOPe benchmark. By default, minlali is defined as nH + nE, where nH and nE are the number of residues assigned to helices and strands, respectively, according to DSSP (Kabsch & Sander, 1983). This reflects the assumption that the common core primarily consists of secondary structure elements.

Due to high redundancy in AFDB2, evaluating every protein in each neighbor shell would be computationally inefficient. Instead, only a representative subset is validated, while redundant proteins are deferred to the final validation round. Representatives are selected using a greedy approach. First, database proteins (targets) are ranked by e‐value relative to their parent protein. Then, targets are processed sequentially: If a target is not masked, it is added to the representative set, and neighboring proteins of the target within a cutoff radius (Foldseek e‐value <1e‐5) are masked to reduce redundancy.

Newly identified neighbors of accepted representatives form the next neighbor shell, and the iteration continues until no new neighbors are found (Figure 1).

In the final validation round, DaliLite is used to evaluate redundant neighbors (Foldseek e‐value <1e‐5) of the accepted representatives, ensuring comprehensive coverage of the fold space.

### 5.4 Annotation

The annotation step crosslinks DaliLite's internal structure identifiers with Uniprot accession numbers and metadata. The mapping is straightforward because AlphaFold Database models were generated from Uniprot sequences.

We assume that the Query structure represents a single domain, and therefore, we expect matches in database proteins to be limited to a single domain. To assign these domains to a Pfam family, we extract the sequence fragments from the stacked structural alignment and run hmmsearch (Eddy, 2011) against the Pfam‐A.hmm library using the trusted cutoff. The Pfam profile with the best e‐value is retained. Since these sequences concatenate fragments representing only the structurally aligned regions, this method may occasionally produce spurious assignments or mismatches that score below the cutoff. However, it avoids the challenges of domain delineation or mapping domain boundaries to gappy alignments and tends to produce correct labels when the alignment covers a sufficiently long region.

### 5.5 Evaluation

To assess search performance, we compare the reported results to the ground truth given by a reference classification.

Denoting the set of reported (query, target) pairs as positives (set P), the ground truth as set TRUE, and their intersection as true positives (set TP = P ∩ TRUE), search performance is assessed by precision (pr) and recall (rc):

1. pr = |TP|/|P|
2. rc = |TP|/|TRUE|

The F1‐score, the harmonic mean of precision and recall, provides an overall measure of performance:

3 F1 = 2 × pr × rc/(pr + rc)

To evaluate search accuracy, we categorized expected outcomes using three suffixes, reflecting the premise that the search aims to retrieve structures with Dali‐validated geometric similarity. Suffix 0 represents the retrieval of all cases from an external reference classification (Pfam or SCOPe). Suffix 1 refines this set to include only *findable* cases—those validated by Dali with *Z* > 2. Suffix 2 applies a stricter criterion to match the criteria set for the common core: requiring at least minimally structurally equivalent residues and significant similarity to the query (*Z*‐score > *zcut*).

For each TRUE set, the corresponding P sets are filtered in a similar manner. Since validation by DaliLite is integrated into the transitive closure algorithm, the user is presented with a filtered result set P_1. To compare transitive closure to direct Foldseek hits, we also ran the Foldseek program using the PDB formatted Query structure as input; here, the raw program output constitutes set P_0.

Since SCOPe follows a hierarchical classification, the TRUE sets were further stratified into three levels. At the fold level, pairs belong to the same fold but different superfamilies. At the superfamily level, pairs belong to the same superfamily but different families. Finally, at the family level, pairs belong to the same family. This stratification ensures a nuanced evaluation of search performance across different degrees of structural similarity.

### 5.6 System architecture

ISS_ProtSci utilizes a client–server architecture. Since most computations, including pairwise Dali alignments, are performed locally on the user's machine, users must download and store protein structure databases for local alignment processing. The transitive closure algorithm, which explores relationships between neighboring structures, depends on a large, centralized knowledge base (KB) of precomputed neighbor relationships. This KB is housed on a remote data server, accessed by ISS_ProtSci over the network. Network queries to the KB are limited to a predefined set with fixed parameters. Figure 7 illustrates the system architecture, highlighting both local and remote dependencies.

### 5.7 Availability

ISS_ProtSci is available from http://ekhidna2.biocenter.helsinki.fi/ISS_ProtSci/. In addition to the distribution package itself, the user must install DaliLite with the AlphaFold Digest database, Foldseek, and hmmsearch. Openmpi is strongly recommended for use with DaliLite, though serial execution of the program is possible. The user manual, at the above link, explains system requirements, installation, configuration, and usage through a worked example. Data for a locally installed version of the sqlite3 databases is available via Zenodo from https://doi.org/10.5281/zenodo.14885122.

## AUTHOR CONTRIBUTIONS

**Hao Liu:** Software; conceptualization; writing – review and editing. **Aleksi Laiho:** Visualization; conceptualization; writing – review and editing. **Petri Törönen:** Writing – review and editing; conceptualization. **Liisa Holm:** Writing – original draft; conceptualization; methodology; writing – review and editing.

## Supporting information


**Data S1.** Supporting Information.

## Data Availability

The data that support the findings of this study are available in Zenodo at https://doi.org/10.5281/zenodo.14885122, reference number 14885122. These data were derived from the following resources available in the public domain: UniprotKB, https://ftp.ebi.ac.uk/pub/databases/uniprot/current_release/knowledgebase/complete/uniprot_*.fasta.gz; AlphaFold Database, https://ftp.ebi.ac.uk/pub/databases/alphafold/v2/; AlphaFold Digestj for DaliLite, http://ekhidna2.biocenter.helsinki.fi/dali/digest.html; Pfam, https://www.ebi.ac.uk/interpro/download/pfam/; Software and benchmark results, http://ekhidna2.biocenter.helsinki.fi/ISS_ProtSci/ISS_ProtSci.tar.gz.
