# Supplementary material for: 3‐D substructure search by transitive closure in AlphaFold database
Source: Protein Sci. 2025 May 22;34(6):e70169. doi: 10.1002/pro.70169 (PMC12095923; doi:10.1002/pro.70169)
Supplement: Supplementary file 1 — Data S1. Supporting Information. [file PRO-34-e70169-s001.pdf]

SUPPLEMENT

Supplementary Table1: Output format.
All *.tsv outputs contain tab-separated values with a header line.
{cd1}.AFDB2.tsv includes columns 1-32 and {cd1}.AFDB2.pf.tsv includes columns 1-35.
Data on gray background is reserved for future use.

Column	Header	Description
1	Rank	Rank of match in descending order of Dali Z-scores
2	Sbjct	Dali identifier of matched structure (sbjct) in AFDB2
3	Database	Query/AFDB. Flags special data for query protein.
4	Z-score	Dali Z-score. Higher values are better.
5	RMSD	Root-mean-square deviation of superimposed CA atoms.
6	Ali-length	Number of structurally equivalent CA atoms in the Dali alignment.
7		
8	Seq-identity	Percentage of identical amino acids of structurally aligned residues.
9	Accession	Uniprot accession number of AlphaFold model
10	Protein-identifier	Uniprot protein identifier of AlphaFold model
11	Sbjct-length	Number of amino acids of sbjct sequence in Uniprot
12	Description	Short description of sbjct protein from Uniprot
13	Taxid	NCBI taxonomy id of sbjct protein
14	Gene	Sbjct protein's gene name from Uniprot
15	Species	Sbjct protein's species from Uniprot
16	Query	Query identifier
17	Query-length	Number of amino acids in query structure
18	Query-coverage	Ali-length divided by Query-length
19	Sbjct-coverage	Ali-length divided by Sbjct-length
20		
21	Foldseek-evalue	E-value of direct Foldseek search with max-seqs 50000
22	qstarts	Start positions of ungapped aligned segments in query
23	sstarts	Start positions of ungapped aligned segments in sbjct
24	lengths	Lengths of ungapped aligned segments
25	rotation	3x3 rotation matrix U
26	translation	translation vector T. UX+T superimposes sbjct X onto query structure.
27	Sbjct-sequence	Amino acid sequence of sbjct
28	Sbjct-dssp	DSSP assignments for sbjct. H = alpha helix, E = beta strand, L = coil
29	sequ-pileup	How sbjct-sequence appears in stacked alignment, ignoring insertions
30	DSSP-pileup	How sbjct-dssp appears in stacked alignment, ignoring insertions
31	dssp-order	Rank of match in seriated view
32		
33	pfam	Pfam identifier of protein family
34	hmmer_evalue	evalue from hmmsearch
35	clan	Pfam clan of protein family. NA if unassigned.

Supplementary Table 2: Counts of Pfam protein families present in selected evaluation data sets.

Case #	TRUE_0	TRUE_1	closure TP_1
1	4	4	4
2	3	3	2
3	7	7	7
4	8	5	5
5	8	8	7
6	26	26	26
7	19	19	17
8	50	50	49
9	134	118	115
10	53	51	48
11	22	17	11
12	31	31	24

Supplementary Table 3: Provisional precision-recall analysis on Pfam benchmark

Series _0: no filtering; series _1: Dali Z-score > 2; series_2: Dali Z-score > 2 and lali > minlali. Precision and recall are provisional, because reference data is positive-unlabelled. Foldseek series have varying e-value cutoff.

method	TRUE_0	TP_0	P_0	TRUE_1	TP_1	P_1	TRUE_2	TP_2	P_2	rc_0	pr_0	F1_0	rc_1	pr_1	F1_1	rc_2	pr_2	F1_2	case
**closure**	15	0	840	14	14	766	13	13	269	0.000	0.000		1.000	0.018	0.036	1.000	0.048	0.092	1
foldseek(1)	15	14	571	14	14	444	13	13	28	0.933	0.025	0.048	1.000	0.032	0.061	1.000	0.464	0.634	1
foldseek(0.1)	15	10	23	14	10	23	13	9	9	0.667	0.435	0.526	0.714	0.435	0.541	0.692	1.000	0.818	1
foldseek(0.01)	15	1	1	14	1	1	13	1	1	0.067	1.000	0.125	0.071	1.000	0.133	0.077	1.000	0.143	1
foldseek(0.001)	15	1	1	14	1	1	13	1	1	0.067	1.000	0.125	0.071	1.000	0.133	0.077	1.000	0.143	1
**closure**	74	0	184	73	72	79	64	64	65	0.000	0.000		0.986	0.911	0.947	1.000	0.985	0.992	2
foldseek(1)	74	72	1497	73	72	78	64	64	65	0.973	0.048	0.092	0.986	0.923	0.954	1.000	0.985	0.992	2
foldseek(0.1)	74	71	1317	73	71	73	64	64	65	0.959	0.054	0.102	0.973	0.973	0.973	1.000	0.985	0.992	2
foldseek(0.01)	74	61	1215	73	61	61	64	61	61	0.824	0.050	0.095	0.836	1.000	0.910	0.953	1.000	0.976	2
foldseek(0.001)	74	60	1106	73	60	60	64	60	60	0.811	0.054	0.102	0.822	1.000	0.902	0.938	1.000	0.968	2
**closure**	186	0	80357	184	183	11752	181	181	5171	0.000	0.000		0.995	0.016	0.031	1.000	0.035	0.068	3
foldseek(1)	186	179	54559	184	179	3752	181	178	2086	0.962	0.003	0.007	0.973	0.048	0.091	0.983	0.085	0.157	3
foldseek(0.1)	186	177	8428	184	177	1078	181	177	726	0.952	0.021	0.041	0.962	0.164	0.281	0.978	0.244	0.390	3
foldseek(0.01)	186	139	2116	184	139	220	181	139	210	0.747	0.066	0.121	0.755	0.632	0.688	0.768	0.662	0.711	3
foldseek(0.001)	186	66	1013	184	66	91	181	66	91	0.355	0.065	0.110	0.359	0.725	0.480	0.365	0.725	0.485	3
**closure**	359	0	1354	342	315	494	300	299	445	0.000	0.000		0.921	0.638	0.754	0.997	0.672	0.803	4
foldseek(1)	359	319	1826	342	317	682	300	285	399	0.889	0.175	0.292	0.927	0.465	0.619	0.950	0.714	0.815	4
foldseek(0.1)	359	286	448	342	284	386	300	261	345	0.797	0.638	0.709	0.830	0.736	0.780	0.870	0.757	0.809	4
foldseek(0.01)	359	207	256	342	207	256	300	205	254	0.577	0.809	0.673	0.605	0.809	0.692	0.683	0.807	0.740	4
foldseek(0.001)	359	140	155	342	140	155	300	138	153	0.390	0.903	0.545	0.409	0.903	0.563	0.460	0.902	0.609	4
**closure**	740	0	8645	719	613	794	719	613	794	0.000	0.000		0.853	0.772	0.810	0.853	0.772	0.810	5
foldseek(1)	740	325	459	719	320	395	719	320	395	0.439	0.708	0.542	0.445	0.810	0.575	0.445	0.810	0.575	5
foldseek(0.1)	740	133	147	719	133	146	719	133	146	0.180	0.905	0.300	0.185	0.911	0.308	0.185	0.911	0.308	5
foldseek(0.01)	740	20	23	719	20	23	719	20	23	0.027	0.870	0.052	0.028	0.870	0.054	0.028	0.870	0.054	5
foldseek(0.001)	740	14	14	719	14	14	719	14	14	0.019	1.000	0.037	0.019	1.000	0.038	0.019	1.000	0.038	5
**closure**	1308	0	40253	1289	1274	13391	1213	1211	2618	0.000	0.000		0.988	0.095	0.174	0.998	0.463	0.632	6
foldseek(1)	1308	1172	4449	1289	1169	3514	1213	1144	1448	0.896	0.263	0.407	0.907	0.333	0.487	0.943	0.790	0.860	6
foldseek(0.1)	1308	1084	1460	1289	1081	1411	1213	1068	1196	0.829	0.742	0.783	0.839	0.766	0.801	0.880	0.893	0.887	6
foldseek(0.01)	1308	932	1023	1289	930	1020	1213	929	1012	0.713	0.911	0.800	0.721	0.912	0.806	0.766	0.918	0.835	6
foldseek(0.001)	1308	752	786	1289	751	785	1213	750	784	0.575	0.957	0.718	0.583	0.957	0.724	0.618	0.957	0.751	6
**closure**	2058	0	36030	1978	1965	9308	1942	1939	5783	0.000	0.000		0.993	0.211	0.348	0.998	0.335	0.502	7
foldseek(1)	2058	1892	1975	1978	1868	1920	1942	1866	1895	0.919	0.958	0.938	0.944	0.973	0.958	0.961	0.985	0.973	7
foldseek(0.1)	2058	1805	1822	1978	1783	1800	1942	1783	1798	0.877	0.991	0.930	0.901	0.991	0.944	0.918	0.992	0.953	7
foldseek(0.01)	2058	1679	1692	1978	1661	1674	1942	1661	1674	0.816	0.992	0.895	0.840	0.992	0.910	0.855	0.992	0.919	7
foldseek(0.001)	2058	1492	1502	1978	1480	1490	1942	1480	1490	0.725	0.993	0.838	0.748	0.993	0.854	0.762	0.993	0.862	7
**closure**	10300	0	33701	9387	9209	13274	5568	5564	6012	0.000	0.000		0.981	0.694	0.813	0.999	0.925	0.961	8
foldseek(1)	10300	3597	3946	9387	3352	3637	5568	2093	2226	0.349	0.912	0.505	0.357	0.922	0.515	0.376	0.940	0.537	8
foldseek(0.1)	10300	661	684	9387	614	636	5568	409	419	0.064	0.966	0.120	0.065	0.965	0.123	0.073	0.976	0.137	8
foldseek(0.01)	10300	66	67	9387	64	65	5568	60	61	0.006	0.985	0.013	0.007	0.985	0.014	0.011	0.984	0.021	8
foldseek(0.001)	10300	59	60	9387	59	60	5568	58	59	0.006	0.983	0.011	0.006	0.983	0.012	0.010	0.983	0.021	8
**closure**	18280	0	78555	10603	10538	12302	10299	10236	11982	0.000	0.000		0.994	0.857	0.920	0.994	0.854	0.919	9
foldseek(1)	18280	1363	1511	10603	1269	1404	10299	1269	1404	0.075	0.902	0.138	0.120	0.904	0.211	0.123	0.904	0.217	9
foldseek(0.1)	18280	168	194	10603	166	192	10299	166	192	0.009	0.866	0.018	0.016	0.865	0.031	0.016	0.865	0.032	9
foldseek(0.01)	18280	2	5	10603	2	5	10299	2	5	0.000	0.400	0.000	0.000	0.400	0.000	0.000	0.400	0.000	9
foldseek(0.001)	18280	0	2	10603	0	2	10299	0	2	0.000	0.000		0.000	0.000		0.000	0.000		9
**closure**	4589	0	8390	4489	4079	5386	3656	3652	3763	0.000	0.000		0.909	0.757	0.826	0.999	0.971	0.984	10
foldseek(1)	4589	2411	8728	4489	2411	8632	3656	2282	2330	0.525	0.276	0.362	0.537	0.279	0.368	0.624	0.979	0.762	10
foldseek(0.1)	4589	2362	4244	4489	2362	4230	3656	2260	2307	0.515	0.557	0.535	0.526	0.558	0.542	0.618	0.980	0.758	10
foldseek(0.01)	4589	2295	3015	4489	2295	3012	3656	2214	2251	0.500	0.761	0.604	0.511	0.762	0.612	0.606	0.984	0.750	10
foldseek(0.001)	4589	2099	2274	4489	2099	2274	3656	2042	2067	0.457	0.923	0.612	0.468	0.923	0.621	0.559	0.988	0.714	10
**closure**	1100	0	2329	720	625	664	718	624	661	0.000	0.000		0.868	0.941	0.903	0.869	0.944	0.905	11
foldseek(1)	1100	222	228	720	222	228	718	221	227	0.202	0.974	0.334	0.308	0.974	0.468	0.308	0.974	0.468	11
foldseek(0.1)	1100	189	189	720	189	189	718	188	188	0.172	1.000	0.293	0.263	1.000	0.416	0.262	1.000	0.415	11
foldseek(0.01)	1100	174	174	720	174	174	718	174	174	0.158	1.000	0.273	0.242	1.000	0.389	0.242	1.000	0.390	11
foldseek(0.001)	1100	164	164	720	164	164	718	164	164	0.149	1.000	0.259	0.228	1.000	0.371	0.228	1.000	0.372	11
**closure**	1753	0	17735	1732	1417	3162	1210	1204	1356	0.000	0.000		0.818	0.448	0.579	0.995	0.888	0.938	12
foldseek(1)	1753	571	638	1732	571	638	1210	517	544	0.326	0.895	0.478	0.330	0.895	0.482	0.427	0.950	0.590	12
foldseek(0.1)	1753	105	111	1732	105	111	1210	97	100	0.060	0.946	0.113	0.061	0.946	0.114	0.080	0.970	0.148	12
foldseek(0.01)	1753	27	27	1732	27	27	1210	27	27	0.015	1.000	0.030	0.016	1.000	0.031	0.022	1.000	0.044	12
foldseek(0.001)	1753	27	27	1732	27	27	1210	27	27	0.015	1.000	0.030	0.016	1.000	0.031	0.022	1.000	0.044	12

Supplementary Table 4: SCOPe test set characteristics.

scope-id	scopclass	nh+ne	nres	t_all	t_fo	t_sf	t_fa	closure_ntest	foldseek_ntest
d1evya1	a.100.1.6	105	158	200	0	185	15	2616	687
d1h54a1	a.102.1.4	284	485	421	256	160	5	1417	373
d1wb9a1	a.113.1.1	213	297	355	0	0	355	1043	989
d3ag3e_	a.118.11.1	68	105	956	956	0	0	53388	53
d2o8pa1	a.118.7.1	184	226	1052	851	0	201	397	843
d1c1ka_	a.120.1.1	151	217	0	0	0	0	6	2
d1sk7a_	a.132.1.2	132	187	67	0	65	2	665	100
d1g8ea_	a.145.1.1	68	98	61	0	0	61	81	72
d1tdza1	a.156.1.2	49	88	440	0	18	422	91256	1385
d1fxka_	a.2.5.1	92	107	1277	1254	0	23	82967	456
d1ffya1	a.27.1.1	180	273	1683	0	0	1683	3309	1780
d1cc5a_	a.3.1.1	39	83	185	0	4	183	74	53
d2sasa_	a.39.1.5	121	185	1074	6	506	794	167	2081
d1hlva2	a.4.1.7	41	63	1368	1353	15	0	282458	49
d1opca_	a.4.6.1	56	99	2032	1921	4	107	116580	435
d1bvsa1	a.5.1.1	29	49	211	47	0	164	895	65
d2fj6a1	a.60.15.1	44	74	413	362	0	51	177	
d1f5qb1	a.74.1.1	96	142	14	0	0	14	2010	811
d3ry4a2	b.1.1.4	55	85	1534	883	640	41	56873	6509
d2rb8a_	b.1.2.1	48	93	2020	2020	0	21	32166	4693
d3cu7a8	b.1.29.1	65	132	1555	1553	2	2	39997	1828
d1jeya1	b.131.1.1	144	281	5	0	0	5	464	320
d2q03a1	b.159.2.1	103	141	0	0	0	0	4135	685
d1tnra_	b.22.1.1	83	144	0	0	0	0	110889	2381
d2piea2	b.26.1.2	53	144	7	0	1	6	37769	731
d3zxfa_	b.29.1.3	77	138	326	0	286	40	102637	4057
d4j9fa_	b.34.2.1	30	62	1708	1585	0	123	112715	2756
d1m5q1_	b.38.1.1	84	127	555	132	234	189	1066	2160
d1nlta2	b.4.1.1	36	80	0	0	0	0	28965	1059
d4crga_	b.47.1.2	110	257	510	0	36	475	5181	1905
d5dgja1	b.47.1.4	100	182	510	0	510	0	5798	1811
d1jjub_	b.69.2.2	177	339	508	503	5	0	44052	15377
d1nr0a2	b.69.4.1	152	299	467	178	21	268	47424	15396
d1ugw.1	b.77.3.1	85	149	23	12	0	11	1753	
d1qrea_	b.81.1.5	92	210	1227	0	1212	29	37750	2399
d1yhfa1	b.82.1.9	63	112	1192	366	824	2	166718	7441
d1yrra1	b.92.1.5	46	85	408	0	398	10	1507	880
d2o2xa1	c.108.1.19	112	215	449	0	428	21	43427	4550
d3a5fa_	c.1.10.1	181	307	16904	14406	911	1587	64046	19712
d1uana_	c.134.1.1	135	220	61	0	0	61	13630	9722
d1i1wa_	c.1.8.3	181	306	16923	15527	1096	300	79608	11103
d2qame1	c.22.1.1	59	201	595	0	0	595	914	919
d2zp1a1	c.26.1.1	194	306	5982	2039	1431	2512	6060	5710
d1u3da2	c.28.1.1	102	187	50	0	0	50	190536	3760
d1ly1a_	c.37.1.1	101	152	7763	0	5986	1777	92884	7972
d1g3qa_	c.37.1.10	148	237	15086	0	13067	2019	33773	11425
d1fpza_	c.45.1.1	106	176	112	0	37	75	28293	4887
d1zgma1	c.47.1.5	46	87	1049	47	537	465	133325	1548
d2aa4a1	c.55.1.10	80	119	5628	3320	2157	151	79980	8378
d4ep4a_	c.55.3.6	111	173	5850	4141	1359	350	19213	8940
d1bd3a_	c.61.1.1	147	224	2217	0	224	1993	1633	3581
d1rjda1	c.66.1.37	222	327	2707	0	2703	4	2931	12331
d1ea5a_	c.69.1.1	283	552	720	0	630	90	976	5523
d2v9la_	c.74.1.1	164	288	127	0	0	127	441	465
d1co3a_	c.82.1.1	313	505	1187	0	138	1049	2147	4029
d1ko7a2	c.91.1.2	109	157	530	0	395	135	675	583
d2y7pa1	c.94.1.1	133	217	1055	0	32	1023	5220	3004
d3v4na2	c.95.1.2	133	218	999	0	403	596	2146	1282
d2hqya2	d.108.1.4	82	139	470	0	456	14	42016	129
d5fafa1	d.109.1.1	56	128	245	74	133	38	23605	786
d1vkka_	d.109.1.2	86	138	259	88	38	133	4319	1110
d1bola_	d.124.1.1	117	222	29	0	0	29	110	102
d1wjwa1	d.129.2.1	48	99	144	89	0	55	136768	
d3tvqa_	d.129.3.6	115	152	302	156	143	3	21747	2719
d1b77a2	d.131.1.2	70	118	91	0	15	76	1728	231
d2pofa1	d.13.1.4	116	220	157	0	118	39	667	878
d1wvfa2	d.145.1.1	117	250	307	0	286	21	1399	1193
d1wfya1	d.15.1.5	23	91	705	421	284	0	35645	
d1oeya1	d.15.2.2	40	77	1385	1385	0	0	36726	4207
d2gf3a2	d.16.1.3	63	104	232	0	160	72	43035	305
d2bi7a2	d.16.1.7	36	69	88	0	77	11	5449	15
d1j34a_	d.169.1.1	65	129	218	0	20	198	26737	1283
d1xg0a_	d.184.1.1	33	77	2	0	0	2	5	5
d4k8va1	d.218.1.15	132	247	81	0	81	0	821	241
d4ixna2	d.237.1.1	62	95	5	0	0	5	155086	1381
d1jx4a1	d.240.1.1	77	101	6	0	0	6	1089	807
d1neia_	d.253.1.1	30	60	5	0	0	5	3843	
d2itka2	d.26.1.1	62	113	696	0	296	400	3687	317

d1w4ta1	d.3.1.5	159	281	52	0	45	7	890	1254
d2hw4a1	d.322.1.1	71	116	34	0	13	21	1354	79
d2guka1	d.360.1.1	81	116	0	0	0	0	3121	385
d3qy3a1	d.38.1.1	80	138	895	0	837	58	13036	2325
d2pu3a_	d.4.1.6	127	216	12	0	0	12	24	20
d4pc3c3	d.43.1.1	91	141	581	0	0	581	940	853
d1kwma2	d.58.3.1	64	95	6341	6302	2	37	244274	3029
d3hf5a1	d.58.4.19	63	126	4795	4569	226	0	212751	6378
d2cz4a1	d.58.5.1	66	99	7143	6879	202	62	62910	11887
d3lqva_	d.58.7.1	59	113	6138	6046	0	92	31545	8305
d2qi2a3	d.79.3.2	71	95	679	367	229	83	1324	1264
d1p1ja2	d.81.1.3	83	115	1901	14	1097	790	6084	960
d3t3la_	d.82.2.1	66	121	157	8	1	148	72705	264
d1vfra_	d.90.1.1	135	217	30	0	0	30	268	246
d3ov1a_	d.93.1.1	40	108	150	0	0	150	41219	993

Supplementary Table 5: External programs and data sources

Name	Version	URL
DaliLite.v5	11 April 2025	http://ekhidna2.biocenter.helsinki.dali/#download
Foldseek	6e0b30cb13ef26a6470a5c2a3e77e6e4ecf2d02e	https://mmseqs.com/foldseek/
hmmsearch	HMMER 3.2.1 (June 2018)	http://hmmer.org/download.html
tbljoin	n.a.	https://github.com/stefan-schroedl/tabulator
UniprotKB	March 2023	https://ftp.ebi.ac.uk/pub/databases/uniprot/current_release/knowledgebase/complete/uniprot_*.fasta.gz
AFDB2	March 2023	https://ftp.ebi.ac.uk/pub/databases/alphafold/v2/
AFDB2 for Dali	21 February 2025	http://ekhidna2.biocenter.helsinki.fi/dali/digest.html
Pfam	October 2021	https://www.ebi.ac.uk/interpro/download/pfam/

Supplementary Table 6. Area under precision-recall curve in SCOPe benchmark.

AUPRC statistic	closure	fs(e<1; zscore)	fs(e<1; evalue)	fs(e<10; evalue)
query-wise average, alpha class	**0.57**	0.38	0.37	0.45
query-wise average, beta class	**0.76**	0.64	0.59	0.64
query-wise average, alpha/beta class	**0.79**	0.67	0.66	0.71
query-wise average, alpha+beta class	**0.76**	0.57	0.51	0.55
query-wise average, all	**0.73**	0.57	0.53	0.59
query-wise median, all	**0.94**	0.61	0.57	0.64
Pooled data	**0.66**	0.38	0.37	0.43

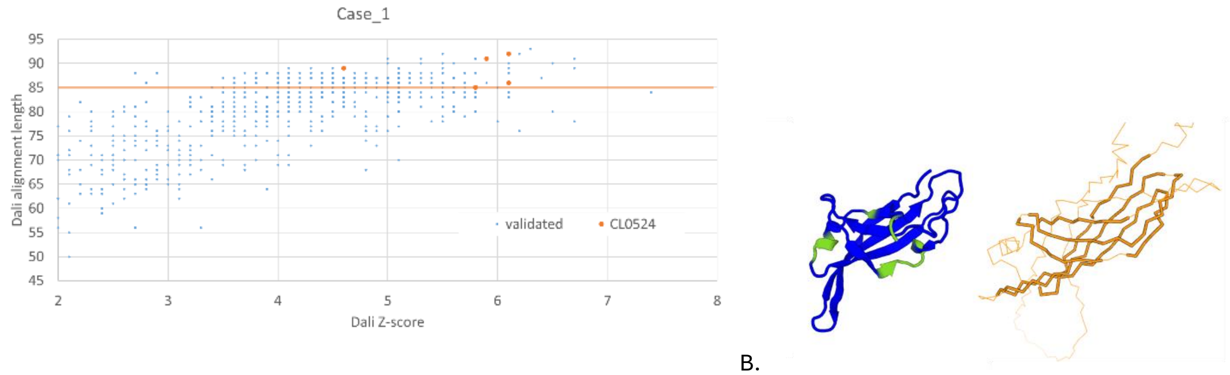

A. B.

Supplementary Figure 1: **An apparent false positive**. (A) Results in the P_1 set of hits by Transitive Closure for Example #1. Many hits exceed the horizontal line at minlali = 85, but their Z-scores are notably lower than those of most positive controls from Pfam clan CL0524. (B) The low Z-scores indicate partial domain overlap between the query structure (PDB: 3sb3, chain A; blue) and target protein (Dali: a9xuA, Uniprot: B4FUU9; orange), yielding a Z-score of 3.9. The 88 structurally equivalent positions are shown by blue colour and thick lines, respectively.

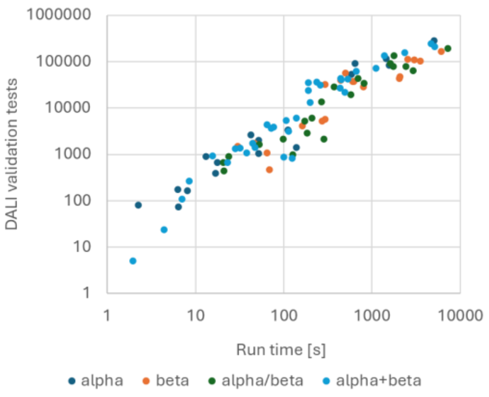

Supplementary Figure 2: Run times of test cases in the SCOPe benchmark. Data points are colored according to SCOPe class. The distribution of run times is fairly uniform on the log scale, and correlates with the number of Dali validation tests.

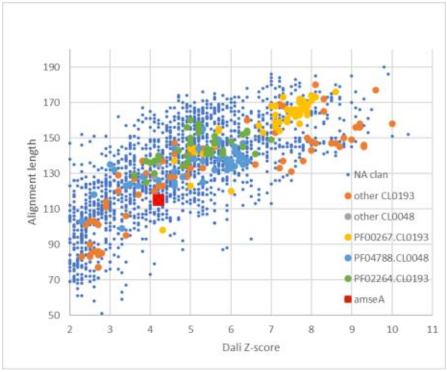

A.

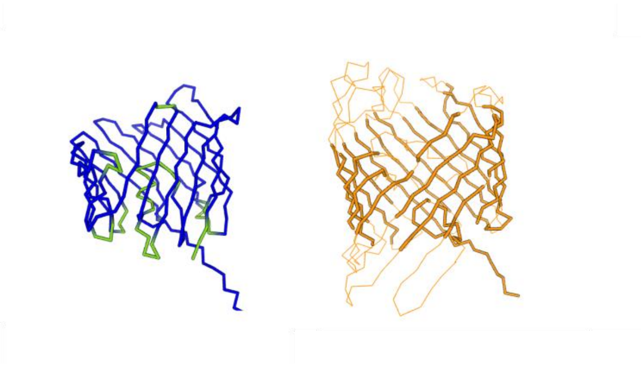

B.

Supplementary Figure 3: **An apparent false negative in the P_2 set for test case #4**. **(A)** Transitive closure search results using a member of family PF06788 as query. The query structure is lj85A (Uniprot: B6IB19) and the minlali parameter had the default value (nH+nE = 140). Figure 6 in main text showed that PF06788 is an outlier in clan CL0395. The red square shows the position of amseA, the query structure used in Figure 6. As shown here, the closest structural neighbors of PF06788 in the AFDB2 database belong to another clan entirely, namely CL0193, and particularly family PF00267 (orange dots). **(B)** Query and target structures (rzfwA, Uniprot: Q01606, a member of PF00267) viewed in the same coordinate frame. The structurally equivalent residues are blue in the query (left) and rendered with thick lines in the target (right). The query and target both have a 16-stranded antiparallel beta-barrel fold, aligning over 184 CA atoms with 4.0 A r. m.s.d. and Z-score of 9.1.
